# Supplementary material for: Gelation Impairs Phase Separation and Small Molecule Migration in Polymer Mixtures
Source: Polymers (Basel). 2020 Jul 16;12(7):1576. doi: 10.3390/polym12071576 (PMC7407309; doi:10.3390/polym12071576)
Supplement: Supplementary file 1 [file polymers-12-01576-s001.zip › Supplementary Materials/polymers-774190-supl.pdf]

Gelation Impairs Phase Separation and Small Molecule Migration in Polymer Mixtures (Supplementary Material)

Biswaroop Mukherjee[†] and Buddhapriya Chakrabarti[‡*]

[†] *Department of Physics and Astronomy,*

University of Sheffield, Sheffield S3 7RH.

(Dated: July 13, 2020)

MESOSCALE DYNAMICS

The free energy functional describing an incompressible binary polymer mixture [1, 2], in two space dimensions, confined between selectively attracting walls (surfaces), located at $z = 0$ and $z = d$ is given by

$$F[\phi(\mathbf{r})] = \frac{1}{a^2}\int_0^d \int_0^d [f_{FH}(\phi) + k(\phi)(\nabla\phi)^2 + f_0(\phi)\delta(z) + f_d(\phi)\delta(z-d)]dxdz, \quad (1)$$

where the first term is the bulk free energy and the second term accounts for energy costs associated with spatial gradients of the composition field, $\phi(\mathbf{r}, t)$. The relevant dynamical equation which governs how the fluid approaches equilibrium, starting from an initial non-equilibrium state, is

$$\frac{\partial\phi(\mathbf{r}, t)}{\partial t} = \nabla \cdot \left[M\nabla\frac{\delta F[\phi(\mathbf{r}, t)]}{\delta\phi(\mathbf{r}, t)} + \theta(\mathbf{r}, t)\right] \quad (2)$$

where M is the mobility, which is assumed to be composition independent and the functional derivative of the coarse-grained free energy functional, $\frac{\delta F[\phi(\mathbf{r},t)]}{\delta\phi(\mathbf{r},t)}$ is the local chemical potential. $\theta(\mathbf{r}, t)$ is the vectorial conserved noise, with a zero mean, where, $\langle\theta_i(\mathbf{r}, t)\rangle = 0$ and the fluctuation-dissipation relation $\langle\theta_i(\mathbf{r}, t)\theta_j(\mathbf{r}', t')\rangle = 2MK_BT\, \delta_{ij}\delta(\mathbf{r} - \mathbf{r}')\delta(t - t')$. The conserved noise is then implemented by computing the divergence of the noise field.

The expression for the chemical potential, at space-point **r**, is given by,

$$\mu(\mathbf{r}) = -2k(\phi)\nabla^2\phi - \frac{dk(\phi)}{d\phi}(\nabla\phi)^2 + \frac{\partial f_{FH}}{\partial\phi} + [+2k(\phi)\nabla\phi + \frac{\partial f_d(\phi)}{\partial\phi}]\delta(z-d) + [-2k(\phi)\nabla\phi + \frac{\partial f_0(\phi)}{\partial\phi}]\delta(z) \quad (3)$$

Here the first two terms, in Eq. 3, are associated with spatial variations of the composition, the third term is associated with the bulk free energy. The last two terms of Eq. 3 are associated with the interaction with the external walls. Upon non-dimensionalising spatial coordinates via $z' = |\chi - \chi_s|^{1/2}z/a$, $x' = |\chi - \chi_s|^{1/2}x/a$ and time via $\tau = N_A M|\chi - \chi_s|^2 t/a^2$ for the polymer-oligomer problem and via the relation $\tau = N_B M|\chi - \chi_s|^2 t/a^2$ for the gel-oligomer problem, the resulting diffusion equation transforms to following non-dimensional form

$$\frac{\partial\tilde{\phi}(x', z', \tau)}{\partial\tau} = \frac{1}{N}\frac{1}{|\chi - \chi_s|}\nabla'^2\tilde{\mu}(x', z', \tau) + \nabla' \cdot \tilde{\theta}(x', z', \tau) \quad (4)$$

where $N = N_A$ for the polymer-oligomer mixture and $N = N_B$ for the gel-oligomer mixture.

The dimension of the noise term, $\theta(\mathbf{r}, t)$, appearing in Eq. 2 is l_0/t_0, where l_0 is equal to $a/|\chi - \chi_s|^{1/2}$ and t_0 is equal to $a^2/NM|\chi - \chi_s|^2$, are the basic units of length and time, respectively.

The the basic unit of noise, θ_0 is equal to $\frac{NM|\chi-\chi_s|^{3/2}}{a^2}$. Upon non-dimensionalizing the noise term, we arrive at a re-scaled fluctuation-dissipation relation, which reads

$$\begin{aligned} \langle\tilde{\theta}_i(\mathbf{r}',t')\tilde{\theta}_j(\mathbf{r}'',t'')\rangle &= \frac{2Mk_BT}{\theta_0^2}\frac{1}{l_0^d t_0}\delta'(\mathbf{r}'-\mathbf{r}'')\delta'(t'-t'')\delta_{ij} \\ &= 2\epsilon\delta'(\mathbf{r}'-\mathbf{r}'')\delta'(t'-t'')\delta_{ij} \end{aligned} \quad (5)$$

where the re-scaled noise amplitude, $\epsilon = \frac{k_BT}{a^d}\frac{1}{N}|\chi-\chi_s|^{(\frac{d}{2}-1)}$, where d is the number of space dimensions. Our simulations are performed in $d = 2$ space dimensions, hence, $\epsilon = \frac{1}{N}$. Thus we generate the noise field by drawing uniformly distributed random numbers between $-A$ and $+A$, where $A = (\frac{\frac{4}{N_A}}{(\Delta x)^2(\Delta\tau)})^{1/2}$. Since the noise appears inside a divergence, we apply a finite difference form for the derivatives appearing in the divergence and then add it to the dynamical equations.

Next we detail how we non-dimensionalize the individual terms contributing to $\tilde{\mu}(z')$ for each individual term appearing in Eq. 3. Since $dz = \frac{a}{|\chi-\chi_s|^{1/2}}dz'$, $\nabla^2\phi$ transforms to $\frac{|\chi-\chi_s|}{a^2}\nabla'^2$ and the factor $|\chi-\chi_s|$ cancels the same factor appearing in the denominator on the R.H.S of Eq. 4. Therefore, this term finally transforms to, $-2\frac{k(\phi)}{a^2}\nabla'^2\phi$. The term $\frac{dk}{d\phi}(\nabla\phi)^2$ similarly transforms to $-\frac{1}{a^2}\frac{dk(\phi)}{d\phi}(\nabla'\phi)^2$. The surface terms of the type $2k(\phi)\nabla\phi\delta(z)$ transforms to $2\frac{k(\phi)}{a^2}\frac{1}{\Delta z'}\delta(z')$. The surface term $\frac{\partial f_S(\phi)}{\partial\phi}\delta(z)$ transforms to $\frac{\partial f_S(\phi)}{\partial\phi}\frac{1}{\Delta z'}\frac{1}{|\chi-\chi_s|^{1/2}a}$.

Incorporating the above transformations, the final expression for $\tilde{\mu}(x',z')$ is given by

$$\begin{aligned} \tilde{\mu}(x',z') &= -2\frac{k(\phi)}{a^2}\nabla'^2\phi - \frac{1}{a^2}\frac{dk(\phi)}{d\phi}(\nabla'\phi)^2 + \frac{1}{|\chi-\chi_s|}\frac{\partial f_{FH}}{\partial\phi} \\ &+ [+2\frac{k(\phi)}{a^2}\nabla'\phi + \frac{1}{|\chi-\chi_s|^{1/2}a}\frac{\partial f_d(\phi)}{\partial\phi}]\frac{\delta(z-d)}{\Delta z'} \\ &+ [-2\frac{k(\phi)}{a^2}\nabla'\phi + \frac{1}{|\chi-\chi_s|^{1/2}a}\frac{\partial f_0(\phi)}{\partial\phi}]\frac{\delta(z)}{\Delta z'} \end{aligned} \quad (6)$$

The computation is being performed on a discretized, square grid (50×50) with $\Delta x' = \Delta z'$. The walls are parallel to the X axis, and are located at $z' = 1$ and $z' = 50$ respectively. For this $2D$ simulation, the diffusion equation for the evolution of the density field is given by,

$$\begin{aligned} \frac{\partial\tilde{\phi}_{ik}}{\partial\tau} &= \frac{1}{N}\nabla'^2\left(\frac{1}{|\chi-\chi_s|}\frac{\partial f_{FH}}{\partial\phi}|_{ik} + \frac{(1-2\phi_{ik})}{\phi_{ik}(1-\phi_{ik})}\frac{k_{ik}}{a^2}(\nabla'\phi|_{ik})^2 - 2\frac{k_{ik}}{a^2}\nabla'^2\phi|_{ik}\right) \\ &+ \frac{1}{N}\nabla'^2\left(\frac{\delta_{k,D}}{\Delta z'}\left[\frac{1}{|\chi-\chi_s|^{1/2}a}\frac{\partial f_D}{\partial\phi_D}|_{iD} + 2\frac{k_{iD}}{a^2}\nabla'_z\phi|_{iD}\right] + \frac{\delta_{k,1}}{\Delta z'}\left[\frac{1}{|\chi-\chi_s|^{1/2}a}\frac{\partial f_0}{\partial\phi_0}|_{i1} + 2\frac{k_{i1}}{a^2}\nabla'_z\phi|_{i1}\right]\right) \end{aligned} \quad (7)$$

The bulk terms and the surface terms have been separated for clarity. The expression inside the ∇'^2 in Eq. 7 is the chemical potential, $\tilde{\mu}(i,k)$, for any point inside the computational lattice. To update the density field, $\tilde{\phi}(x',z')$, we have to compute $\nabla'^2\tilde{\mu}(x',z')$, which requires one to define the values of the fields, $\tilde{\mu}(x',z')$ and $\tilde{\phi}(x',z')$ outside the computational lattice. The fictitous points, bordering the square computational region, have the following equations : (a) $k=0$ and i varies between 1 to 50 (b) $k=51$ and i varies between 1 and 50, (c) $i=0$ and k varies from 1 to 50 and (d) $i=51$ and k again varies from 1 to 50.

To obtain $\tilde{\phi}(x',z')$ at these fictitous points we employ the following numerical scheme [2]. The discretized version of the gradient along Z, according to the central difference scheme, computed at $k=1$, is given by

$$(\nabla_c\tilde{\phi})_{i,1} = \frac{\tilde{\phi}(i,2)-\tilde{\phi}(i,0)}{2\Delta z'} \tag{8}$$

Similarly the gradient computed by the forward difference scheme at $k=1$, is given by

$$(\nabla_f\tilde{\phi})_{i,1} = \frac{-3\tilde{\phi}(i,1)+2\tilde{\phi}(i,2)-\tilde{\phi}(i,3)}{2\Delta z'} \tag{9}$$

Equating the two gradients enable us to solve for $\phi(i,0)$ and the resulting expression for $\tilde{\phi}(i,0)$ is

$$\tilde{\phi}(i,0) = 3\tilde{\phi}(i,1) - 3\tilde{\phi}(i,2) + \tilde{\phi}(i,3) \tag{10}$$

Similarly for the boundary at $k=N_z$ we can get the values of the field at the fictitious nodes by using the equation,

$$\tilde{\phi}(i,N_z+1) = 3\tilde{\phi}(i,N_z) - 3\tilde{\phi}(i,N_z-1) + \tilde{\phi}(i,N_z-2) \tag{11}$$

The chemical potential at the fictitious points are computed via invoking a "no flux" boundary condition at the walls, which ensures that the total material is conserved during the temporal evolution of the density fields. Thus we impose $J_z|_{i,1} = J_z|_{i,N_z} = 0$, which translates to

$$\begin{aligned}\tilde{\mu}(i,0) &= \tilde{\mu}(i,1)\\ \tilde{\mu}(i,N_z+1) &= \tilde{\mu}(i,N_z)\end{aligned} \tag{12}$$

Additionally we impose periodic boundary conditions along the x direction, which implies

$$\begin{aligned}\tilde{\phi}(0,k) &= \tilde{\phi}(N_x,k)\\ \tilde{\phi}(N_x+1,k) &= \tilde{\phi}(1,k)\\ \tilde{\mu}(0,k) &= \tilde{\mu}(N_x,k)\\ \tilde{\mu}(N_x+1,k) &= \tilde{\mu}(1,k)\end{aligned} \tag{13}$$

With this the composition and chemical potential fields have been specified at all computational and also the fictitious points. Now we update the composition field via a forward Euler method, where,

$$\tilde{\phi}_{i,k}^{new} = \tilde{\phi}_{i,k}^{old} + ((\nabla'^2 \tilde{\mu})_{i,k} + (\nabla' \cdot \tilde{\theta})_{i,k})\delta t \tag{14}$$

where $\tilde{\phi}_{i,k}^{new}$ and $\tilde{\phi}_{i,k}^{old}$ are the updated and the old values of the composition field at a specified lattice point. With the updated composition fields obtained at all the computational points one obtains the values of the fields at the fictitious points by the above prescription. Then one computes the updated values of the chemical potential field at the computational points from the RHS of Eq. 7 and then one calculates the chemical potential at the fictitious points by a method discussed above. The above operations are then repeated iteratively to simultaneously evolve the composition and the chemical potential fields, till equilibrium is established, which means the equalisation (within numerical precision) of the chemical potential at all space points.

MOLECULAR DYNAMICS SIMULATIONS

We perform molecular dynamics (MD) simulations of a mixture of (a) polymers (8-mers) and oligomers (4-mers) and (b) an end-grafted gel and oligomers (4-mers), which are confined between two planar walls, oriented perpendicular to the z axis of the simulation box. The upper wall selectively attracts the oligomers, while the lower wall is neutral to both species. We observe the surface-directed coarsening of the mixture as it is quenched instantaneously from a high to a low temperature. The polymer and the oligomer chains are modelled as flexible bead-spring chains of Lennard-Jones particles of diameter σ. The polymer chains of species A are made up of 8 beads, whereas, the B oligomers are made up of 4 beads. The potential between any pair of monomers at a separation of r = $\| \mathbf{r_i} - \mathbf{r_j} \|$ is

$$\begin{aligned} U(r) &= V_{LJ}(r) - V_{LJ}(r_c) - (r - r_c)(\frac{dV_{LJ}}{dr})_{r=r_c}, r < r_c \\ &= 0, r > r_c \end{aligned} \tag{15}$$

In Eq. 15 the interaction, $V_{LJ}(r)$ is given by,

$$V_{LJ}(r) = 4\epsilon_{\alpha\beta}\left[(\frac{\sigma}{r})^{12} - (\frac{\sigma}{r})^{6}\right] \tag{16}$$

is the standard Lennard-Jones (LJ) potential and r_c, which is chosen as 2.5σ, is the cut-off distance beyond which the interaction is set to zero. The indices α and β denote the binary species A and

B and the subtraction of V_{LJ}(r) and the linear term proportional to $(r - r_c)$ makes the potential and the force continuous at the cutoff separation. The LJ energies are chosen to be

$$\epsilon_{AA} = \epsilon_{BB} = 2\epsilon_{AB} = \epsilon \tag{17}$$

which mimics a liquid mixture, that is prone to phase-separate. In addition to the above non-bonded interactions, the nearest neighbours along a polymer chain also interact via the anharmonic finitely-extensible non-linear elastic (FENE) potential

$$V_{bond}(r) = -\frac{1}{2}kR_0^2 \ln\left[1 - (\frac{r}{R_0})^2\right] \tag{18}$$

In the above equation, k sets the energy scale and R_0 sets the range of the bond potential. We have set $k = 40\epsilon/\sigma^2$ and $R_0 = 1.5\sigma$, a choice which ensures that phantom bond crossings and chain breakings are eliminated. All the simulations have been performed by GROMACS and the values chosen for $\sigma = 0.339nm$, while $\epsilon = 0.359kJ/mol$ and the value of masses of all the beads have been chosen as $m = 12.01amu$. These parameters correspond to that of the carbon atom. However, these values do not have any special significance and any other set of parameters would have worked as well. All simulation results have been expressed in dimensionless quantities: lengths have been expressed in units of σ, times have been expressed in units of $\tau_{LJ} = \sqrt{\frac{m\sigma^2}{\epsilon}} \approx 2ps$ for the above values of the parameters, and temperatures have been quoted in dimensionless units of $T^* = k_BT/\epsilon$. The timestep used for performing the MD simulations were $\Delta\tau = 4fs = 0.002\tau_{LJ}$. The modified LJ potential described in Eq. 15 has been tabulated with a spacing of $\Delta r = 0.002nm$. The upper and the lower walls consist of atoms of size σ, same as those of the oligomers and polymers beads, arranged in a square lattice of side equal to R_0, which were held in their positions via the application of stiff, harmonic position restraints. The interaction between the upper wall and monomers of type A is repulsive, while interactions with monomers of type B is of the type described by Eq. 15 with strength equal to ϵ. The lower wall interacts with both monomers via a repulsive interaction. The repulsive interaction is a Weeks-Chandler-Andersen (WCA) type shifted LJ interaction, truncated at the minima, $2^{1/6}\sigma$, and shifted upwards so that the new minima is at zero energy.

The total number of particles were chosen in such a fashion that the dimensionless density, $\rho^* = N\sigma^3/(L_xL_yL_z) \approx 1$ for all our simulations. This choice ensures that the system is liquid and is far from solid-liquid and liquid-gas transitions at the simulated temperatures. The initial states for the quench simulations were generated by performing a long MD simulation at $T^i = 10$. Five

different configurations from this high temperature trajectory were chosen as initial configurations for the quench simulations. An instantaneous quench was performed by setting the temperature of the system at the final value of $T_f = 1$ and 1.875 and the simulations were run for, τ/τ_{LJ} = 10^5 time steps. Various measured quantities were averaged over trajectories obtained from five independent starting configurations. The quench simulations were performed in a constant volume ensemble and the temperature was maintained by a Nose-Hoover thermostat, which is known to preserve hydrodynamics. The composition of the mixture, simulated in the MD simulations, was highly off-critical, with the oligomers accounting for approximately ≈ 10 %, with the rest of the molecules being the longer polymers in the first situation and the end-linked gel in the second set of simulations. The gel was prepared by analysing the starting configurations at $T^i = 10$ and by introducing additional FENE bonds between the end beads of polymer A, which were within a cut-off distance, R_0. The quench simulations were then performed with the modified topology for each independent starting configuration.

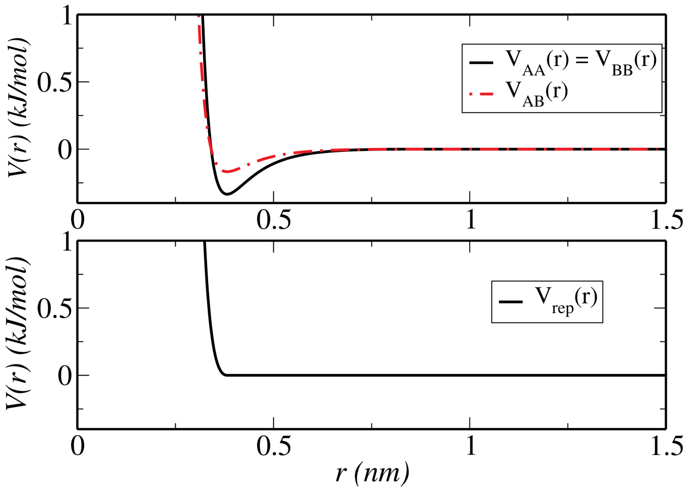

FIG. 1: The potential functions for various interaction potentials used in the MD simulations are shown in the above figure.

COMPARISON OF METHODS

Figure 2 compares the snapshots obtained from MD simulations, for quench to $T_f = 1$, at long time ($\tau = \tau_{LJ} \times 10^5$) after the quench in a polymer-oligomer mixture (left panel) and a gel-oligomer mixture (right panel), confined between a an oligomer attracting wall at the top and a neutral wall below. It is clear that the phase separation is more complete, with a wide depletion

region following the wetting layer and clearly demarcated polymer rich and oligomer rich domains appearing on the left panel. Compared to that, the gel-oligomer system shows several small clusters of oligomer rich domains and a much narrow depletion region. The spatial variation, with distances measured down from the upper wall, of the local composition of the oligomers, in the two mixtures, averaged over the two lateral dimensions parallel to the walls, is shown in panels (a) and (b) of Figure 4 in the main manuscript. This clearly shows a more pronounced wetting layer, of width $\sim 4\sigma$, in the polymer-oligomer system, compared to the gel-oligomer system. The difference in surface migration, which is more pronounced for the quench to T* = 1, reduces for the quench to $T_f = 1.875$. One can also explicitly compute the time-series for the number of oligomers that have migrated to the wetting layer (within a distance of $\approx 4\sigma$ from the upper wall. Figure 3 shows the time-evolution of the average fraction of the migrant molecules for mixtures quenched to $T_f = 1$ (upper panel) and one quenched to $T_f = 1.875$ (lower panel). The filled circled denote the migrant fraction in the polymer-oligomer systems, while the filled squares denote the same for the gel-oligomer mixtures. The mean values and the error bars have been computed over five independent quench simulations for each temperature. The time-evolution of the migrant faction grows initially with time and it then saturates at long times, which occurs due to the presence of a finite number of oligomers in the vicinity of the adsorbing upper wall. The presence of several oligomer clusters in the vicinity of the adsorbing upper wall in the gel-oligomer mixture (see right panel of Figure 2) exhibits arrested domain growth. This arrest arises due to the presence of end-links for the gel, which impedes the growth of density fluctuations, resulting from the temperature quench. Owing to the presence of these end-links, the gel behaves as a significantly larger molecule, with much longer relaxation times compared to the density-relaxation timescale of the oligomer clusters. Dynamical assymetry between components of phase-separating mixtures are known to lead to novel coarsening phenomena and "non-classical" coarsening exponents, differing from the Lifshitz-Slyozov coarsening. It is clear that the disparity of fraction of migrant molecules at long times in the wetting layer of polymer-oligomer mixture shown in Figure 3, is approximately twice compared to that in the gel-oligomer mixtures for quench to $T_f = 1$. This aspect agrees with what had been observed earlier via SCFT , where it was found that surface migration in polymer mixtures can be tuned by including elastic interactions, as gels posess non-zero bulk moduli, with lesser surface migration as one increases the bulk modulus of the gel. These results were thermodynamics in nature and do not say anything about how the system evolves from the initial non-quilibribum state to the

eventual equilibrium. Compared to the quench to $T_f = 1$, for the quench to $T_f = 1.875$, the disparity in the migrant fraction in the polymer-oligomer and the gel-oligomer simulations are much less. This is understandable, as enhanced diffusion at higher temperatures enables the phase separating oligomers to overcome the geometrical constraints posed by the end-links of the gel.

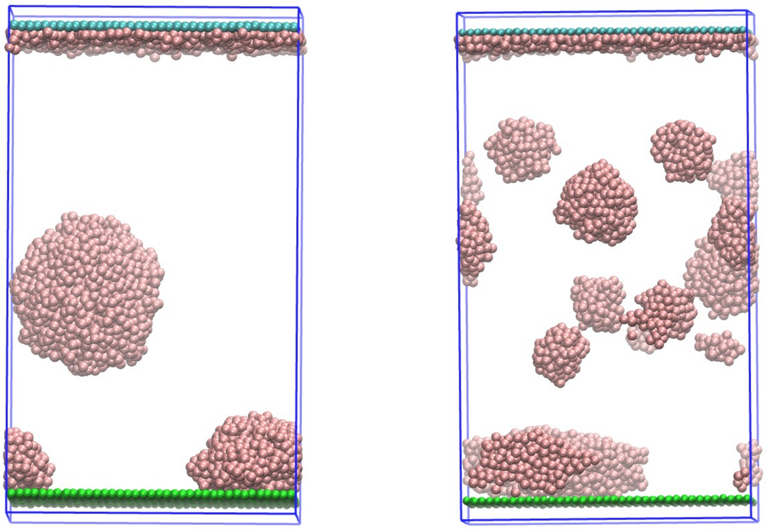

FIG. 2: $CGMD$ simulation snapshot at time $\tau/\tau_{LJ} = 10^5$ and temperature $T_f = 1$, of surface directed (upper surface attracts the oligomers) phase separation in polymer-oligomer mixtures on the left and a gel-oligomer mixture on the right. Only the oligomers, which are the minor component, are highlighted for clarity.

Fig.3 shows the surface concentration of migrant molecules obtained from $CGMD$ simulations for a polymer-oligomer and gel-oligomer mixture when quenched from an initial temperature $T_i = 10$ to final temperatures $T_f = 1$ panel (a) and $T_f = 1.875$ panel (b). As seen from the figure a deeper quench results in a stronger phase-separation arrest leading to a smaller surface fraction of migrants both for oligomer-polymer and oligomer-gel systems.

To rationalise these results and to have access to larger length-scales and longer time-scales, we have also studied the above problem of surface migration following a temperature quench, in a meso-scale description of polymer mixtures in an assymetric confinement between two walls. Figure 3 of the main manuscript shows the coarsening phenomena in a 70:30 polymer-oligomer mixture, quenched to χ_{sim} = 1.1 χ_{sp}, in presence of assymetric confinement with one wall having preferential attraction for the oligomers (shown in dark colour), while the other wall is neutral.

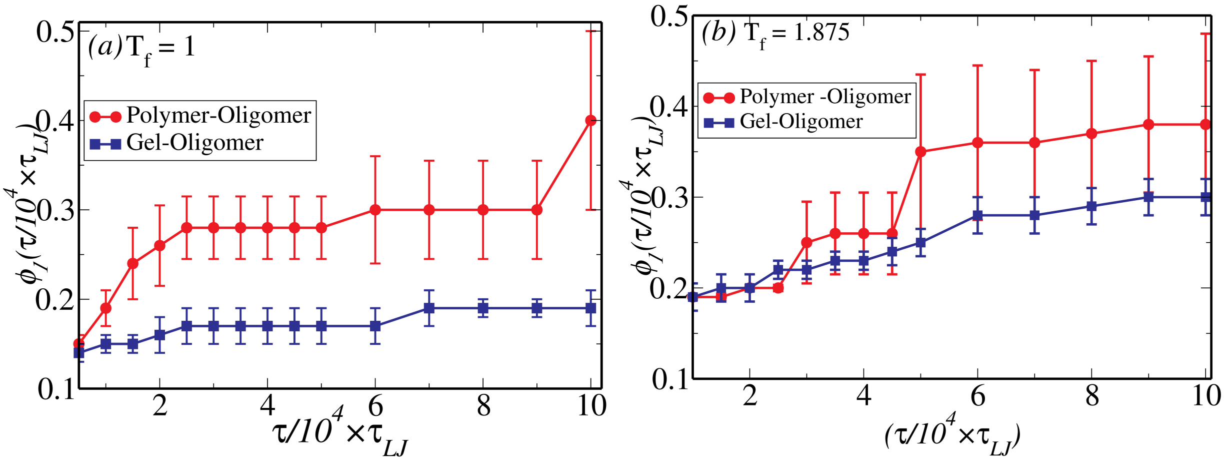

FIG. 3: The fraction of migrant molecule computed from an MD simulation of a polymer-oligomer mixture and a gel-oligomer mixture is shown in panel (a) for an initial temperature $T_i = 10$ to final temperature $T_f = 1.875$, while panel (b) shows the surface migrant fraction at $T_f = 1$.

We observe the development of a wetting layer, rich in oligomers, near upper the adsorbing wall and coarsening in the bulk. Adjoining the flat wetting layer, a depletion region develops, with $\phi \sim 0.9$ and with significant extent, and also the condensation of a sizeable fraction of oligomers on the neutral surface. These aspects are qualitatively similar to what has been observed in the MD simulations of polymer-oligomer mixtures (see Figure 2). In addition to this we also observe coarsening in the bulk (in regions away from the confining walls) and condensation of a significant fraction of oligomers on the neutral walls at long times. Compared to the polymer oligomer mixtures, the phase separation and surface migration in gel-oligomer mixtures is significantly suppressed. From Figure 3 (panel (b)) of the main manuscript, we observe that in gel-oligomer mixtures, lower values of composition field, ϕ, become more costly with increasing values of the gel's bulk modulus, B. On the other hand oligomer-adsorbing surface prefers lower values of ϕ. This competition drives the surface wetting and phase separation in the gel-oligomer mixtures.

Figure 4 shows the time evolution of the wetting layer computed from mesoscale simulations of a polymer-oligomer mixture (filled red circles), a gel-oligomer mixture with bulk modulus, $B = 0.05$ (filled blue squares) and another gel-oligomer mixture with $B = 0.1$ (filled blue diamonds). It is known, for deep quenches, the surface growth in symmetric polymer mixtures exhibit a LSW growth law with temporal dependence, $\ell_w(\tau) \sim \tau^{1/3}$. We observe a similar surface-growth exponent of 0.36, from our mesoscale simulations of assymetric polymer-oligomer mixtures shown in Figure 4. With increasing bulk modulus, we observe reduced surface migra-

tion (see Figure 5 in the main manuscript) and a complete arrest in surface growth occurring at $B = 0.1$, as shown in Figure 4.

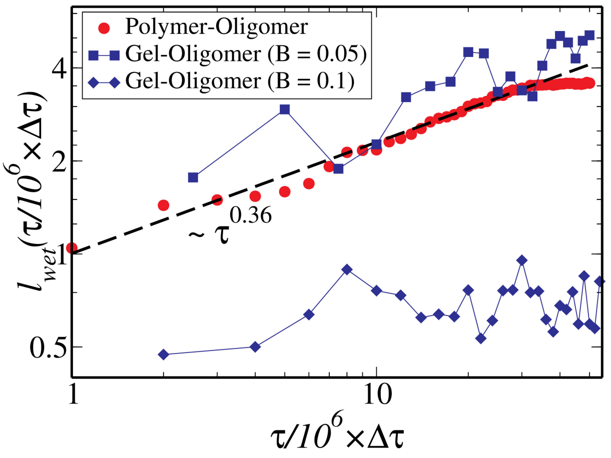

FIG. 4: The temporal dependence of the growth of wetting layer, computed from mesoscale simulations of polymer-oligomer mixture (filled red circles), a gel-oligomer mixture with bulk modulus, $B = 0.05$ (filled blue squares) and another gel-oligomer mixture with $B = 0.1$ (filled blue diamonds). The surface growth in the polymer-oligomer mixture, exhibits a growth law with temporal dependence, $\ell_w(\tau) \sim \tau^{0.36}$, an exponent that is close to the LSW, domain coarsening exponent of $1/3$.

* Electronic address: `b.chakrabarti@sheffield.ac.uk`

[1] Coveney, S.; Clarke, N. Breakup of a Transient Wetting Layer in Polymer Blend Thin Films Unification with 1D Phase Equilibria. *Phys. Rev. Lett.* **2013**, *111*, 125702.

[2] Coveney, S.; Clarke, N. Lateral phase separation in polymer-blend thin films Surface bifurcation. *Phys. Rev. E* **2014**, *89*, 062603.
